# Astrocyte-derived exosomes enriched with miR-873a-5p inhibit neuroinflammation via microglia phenotype modulation after traumatic brain injury

**DOI:** 10.1186/s12974-020-01761-0

**Published:** 2020-03-19

**Authors:** Xiaobing Long, Xiaolong Yao, Qian Jiang, Yiping Yang, Xuejun He, Weidong Tian, Kai Zhao, Huaqiu Zhang

**Affiliations:** 1 Department of Neurosurgery, Tongji Hospital, Tongji Medical College, Huazhong University of Science and Technology, Wuhan, 430030 China; 2 Department of Neurosurgery, First Affiliated Hospital of Medical College, Shihezi University, Shihezi, China

**Keywords:** Exosome, Traumatic brain injury, Microglia, Astrocyte, M1/M2, miR-873a-5p

## Abstract

**Background:**

The interaction between astrocytes and microglia plays a vital role in the damage and repair of brain lesions due to traumatic brain injury (TBI). Recent studies have shown that exosomes act as potent mediators involved in intercellular communication.

**Methods:**

In the current study, the expression of inflammatory factors and miR-873a-5p in the lesion area and oedema area was evaluated in 15 patients with traumatic brain injury. Exosomes secreted by astrocytes were detected by immunofluorescence, Western blot and electron microscopy. A mouse model of TBI and an in vitro model of LPS-induced primary microglia were established to study the protective mechanism of exosomes from miR-873a-5p overexpressing in TBI-induced nerve injury.

**Results:**

We discovered that exosomes derived from activated astrocytes promote microglial M2 phenotype transformation following TBI. More than 100 miRNAs were detected in these astrocyte-derived exosomes. miR-873a-5p is a major component that was highly expressed in human traumatic brain tissue. Moreover, miR-873a-5p significantly inhibited LPS-induced microglial M1 phenotype transformation and the subsequent inflammation through decreased phosphorylation of ERK and NF-κB p65. This effect also greatly improved the modified neurological severity score (mNSS) and attenuated brain injury in a strictly controlled cortical impact mouse model.

**Conclusions:**

Taken together, our research indicates that miRNAs in the exosomes derived from activated astrocytes play a key role in the astrocyte-microglia interaction. miR-873a-5p, as one of the main components of these astrocyte-derived exosomes, attenuated microglia-mediated neuroinflammation and improved neurological deficits following TBI by inhibiting the NF-κB signalling pathway. These findings suggest a potential role for miR-873a-5p in treating traumatic brain injury.

## Introduction

Traumatic brain injury (TBI) has always been a major cause of death and disability worldwide [1]. Due to the complex pathophysiological mechanisms of TBI, effective pharmacotherapy is still lacking. A number of studies have identified that neuroinflammation following TBI plays an important role in secondary pathophysiological damage which might have a more serious and profound influence compared with that of the primary injury.

Microglia are the resident immune cells that initiate inflammation and immune response. Microglia are activated, migrate to the injury site rapidly and play a crucial role in neuroinflammation following TBI [2]. Recent studies have discovered that the role of microglia in TBI is a double-edged sword based on their two polarizations: the pro-inflammatory (M1-like) phenotypes and anti-inflammatory (M2-like) phenotypes [3]. Regulating the polarization of microglia, especially promoting the M2 phenotype transformation following TBI, may be a possible therapeutic strategy. Although some molecules have been identified as selectively increasing M2 polarization [4], such as IL-4, IL-10 and TGF-β, effective medicine for the clinic is still lacking.

Astrocytes are the most abundant cell type in the central nervous system and play a crucial role in maintaining microenvironment stability and neural circuit function [5–7]. Previous studies demonstrated that astrocytes and microglia are active participants in various pathological conditions including brain trauma. Activated astrocytes produce many regulatory factors that influence CNS immunity and provide negative feedback to activated microglia [8, 9].

Recently, exosomes, a type of extracellular vesicles, have been identified as signalling conveyors in cell-to-cell communication [10]. Via cargo proteins, RNAs and miRNAs, exosomes act as key players in triggering, transferring and regulating immune responses to neighbouring cells [11]. Most cells in the CNS have been reported to secrete exosomes into the extracellular environment [12]. Although some studies show that astrocyte-derived exosomes are powerful mediators involved in neuronal plasticity, immune response and neuronal survival under multiple pathological conditions [13], little is known about their function in microglial activation and their role in mediating neuroinflammation after brain trauma.

In the present study, we used an in vitro TBI model to investigate the composition of astrocyte-derived exosomes and their effect on microglial activation, which could lead us to further understand the crosstalk between microglia and astrocytes. Furthermore, the potential functional miRNAs were investigated in a mouse TBI model in vivo to explore a new therapeutic target for neuroinflammation-associated injury.

## Methods

### Animal TBI model and experimental grouping

All experimental procedures were conducted following the existing rules of Huazhong University of Science and Technology and the National Institutes of Health Guidelines for the Care and Use of Laboratory Animals. Male adult mice (C57BL/10ScNJ; age, 10–12 weeks; weight, 20–22 g) were obtained from Huazhong Keji Co. All animals were housed in a controlled environment (temperature, 22 ± 3 °C, under a 12-h light/dark cycle) and were provided standard rodent nutrition and water. The mouse model of TBI was induced according to previous reports [14]. Anaesthesia was surgically induced with chloral hydrate (400 mg/kg body weight) administered intraperitoneally (i.p.), and then the mice were subjected to a unilateral, moderately controlled cortical impact (CCI) of 2.0 mm depth at 3.5 m/s and 500 ms dwell time using the TBI 0310, a pneumatic impacting device (Precision Systems and Instrumentation, Fairfax Station, VA) with a hard stop Bimba cylinder (Bimba Manufacturing, Monee, IL). The size of the bevelled impactor was 5 mm. All craniotomies were placed midway between the bregma and lambda sutures in the left hemisphere of the brain. A total of 100 animals were randomly divided into 4 groups: sham group (*n* = 20), sham + miR-873a-5p agomir group (*n* = 20), TBI group (*n* = 30), and TBI + miR-873a-5p agomir group (*n* = 30). All investigators were blinded to the treatment groups during animal surgery, data collection and analysis.

### Primary microglia and astrocyte culture

Primary microglia and astrocytes were obtained and purified from C57BL/6 mice on postnatal days 1 to 2 as previously described [14]. Briefly, the cerebral cortex was collected and cut into 1-mm^3^ pieces. After digestion with 0.25% trypsin and DNase for 10 min, the cell suspensions were passed through a 70-mm Nylon mesh, and the digestion was ended with DMEM supplemented with 10% FBS and 1% penicillin/streptomycin. These cells were collected by centrifugation and seeded into a culture flask. Microglia within the astrocyte monolayer were removed by shaking at 220 rpm for 40 min after 10 days and were then re-cultured in 6- or 24-well plates. The remaining mixed glial cells in the flask were shaken at 220 rpm for 18 h continuously to remove oligodendroglia, and then the remaining cells in the flask were astrocytes with over 90% purity. Astrocytes and microglia were cultured at 37 °C in a 5% CO_2_ atmosphere for a subsequent experiment.

### Clinical specimen collection and ethics statement

The present study was conducted in accordance with the Declaration of Helsinki and was approved by the Research Ethics Board of Tongji Hospital. Written informed consent was obtained from all individuals who were included in the study. TBI patients were diagnosed according to the World Health Organization criteria. The clinical specimens of damaged brain tissue were taken from 15 patients who were operated on in the neurosurgical emergency department of Tongji Hospital (additional table 1). These tissues were either necrotic brain tissue or severe oedema around the lesion that needed to be removed. The expression of miR-873a-5p in brain tissue was detected by quantitative real-time PCR.

### Cell transfection of the miR-873a-5p mimic and intracerebroventricular infusion

The miR-873a-5p mimic, negative control mimic (NC mimic) and agomir were purchased from RiboBio (China). They were dissolved and diluted according to the instructions provided by the manufacturer. The microglia were transfected with 100 nM aliquots of either the miR-873a-5p mimic or NC mimic using RiboFECT™ CP (RiboBio, China) as per the manufacturer’s protocol. After dissolving and mixing the miR-873a-5p agomir, it was allowed to stand for 15 min at room temperature and was then used for lateral ventricle injection. A Hamilton syringe (Gaoge, China) was inserted at 0.5 mm posterior and 1.0 mm lateral to the bregma and 3.0 mm ventral to the skull under the guidance of a stereotaxic instrument (RWD Life Science). A single dose of miR-873a-5p agomir (5 nM) was infused into the lateral ventricle 20 min after CCI.

### Brain extracts

Brain extracts (Ext) were made as previously described [15]. Briefly, TBI was induced in C57BL/6 mice, and after 1 day, the cortices were collected before they were surgically induced with chloral hydrate (400 mg/kg body weight) for anaesthesia. A total of 4 ml complete medium per cortex were added into glass tubes, and the supernatant was collected after each cortex was ground fully with a glass grinding rod and centrifuged at 1000 rpm for 10 min. Then, 1 ml of the supernatant was aliquoted in 1.5 ml EP tubes and kept at − 80 °C for usage.

### Astrocyte-derived exosome isolation and transmission electron microscopy analysis

The astrocyte-derived exosome isolation procedures were performed at 4 °C as described in the literature [16]. Briefly, supernatants collected from cultured astrocytes were first filtered through a 0.2-μm filter to remove the large debris and dead cells. Small cell debris was removed by centrifugation at 10,000*g* for 30 min, and then the supernatants were recentrifuged at 100,000*g* for 3 h. The supernatants generated at this step were stored at 4 °C for future use as exosome-free controls (the average storage time was no more than 1 week). The pellets were resuspended in 30–50 μl of PBS and stored at − 80 °C for another usage. For the transmission electron microscopy (TEM) morphology investigation, the pellets obtained as described above were subjected to uranyl acetate negative staining on For-mvar carbon-coated 400 mesh copper electron microscopy grids (FCF400-Cu, Electron Microscopy Sciences, Hatfield, PA). Twenty microliters of the sample was applied to the grid and incubated for 5 min at room temperature, and then the excess solution on the grid was wicked off and dried for 30 min with filter papers. An equal part of 10% uranyl acetate was added to the grid for negative staining for 5 min. The preparations obtained were examined at 70 kV with a Philips 208 electron microscope (Philips, Bothell, WA) with an AMT digital imaging system (Advanced Microscopy Techniques Corp., Woburn, MA). Protein concentrations of exosome preparations were determined using the BSA assay. For neural cell treatment with astrocyte exosomes, we diluted the collected exosome-enriched fractions of the stored supernatant as noted above, and the supernatant without exosomes was used as a control. These media were then added to the cultured neural cells.

### Exosome labelling and uptake

Exosomes were fluorescence-labelled with PKH26 (Sigma-Aldrich), a lipophilic dye, according to the manufacturer’s protocol. Exosomes were incubated in diluent C and PKH26 for 5 min at room temperature. PKH26-labelled exosomes were diluted with PBS and ultracentrifuged at 150,000*g* 4 °C for 70 min to enable removal of unincorporated dye contamination from exosome labelling reactions. Subsequently, the PKH26-labelled or denatured exosomes were incubated with primary microglia for 24 h. After the cells were fixed, we stained the cells with 4′,6-diamidino-2-phenylindole (DAPI) and phalloidin (Sigma-Aldrich). The images were obtained under a confocal microscope.

### miRNA microarray analysis

The miRNA microarray analysis was performed by GeneChem (Shanghai, China). The samples of exosomes derived from astrocytes were divided into 2 groups: the Ext group and the CON group, which were with or without exposure to brain extracts, respectively. The quality and integrity of the RNA extracted from the exosomes were evaluated first. Next, 200 ng of total RNA was labelled with the GeneChip 39 IVT Express Kit (Thermo Fisher Scientific) and hybridized to the GeneChip miRNA 3.0 Array (Thermo Fisher Scientific), which covered 1188 mature mouse miRNAs and 889 pre-miRNAs. RNA molecules were then polyadenylated, followed by a ligation step with a biotin-labelled DNA molecule attached. The labelled RNA was finally washed and stained in the GeneChip Fluidics Station 450 and scanned in the GeneChip Scanner 3000 (Thermo Fisher Scientific).

### Western blot analysis

Protein concentrations of medium supernatant, cells and animal tissue were determined by using a BSA kit, and then the protein samples were diluted with 5× sample buffer solution, separated by electrophoresis in a 12% separation gel for 90 min and blocked with 1× PBS containing 5% (w/v) non-fat dried milk (PM) for 1 h at room temperature. Then, the cells were incubated with primary antibodies (iNOS, 1:1000; HMGB1, 1:1000; IL-1β, 1:1000; Arg1, 1:1000; CD9, 1:1000; CD63, 1:1000; p-NK-κB, 1:1000; NK-κB, 1:1000; IL-4, 1:1000; and IL-10, 1:1000—Abcam, USA; MyD88, 1:1000; p-ERK, 1:1000; and ERK, 1:1000—Cell Signaling Technology, Beverly, MA, USA) at 4 °C overnight. The membranes were then washed and incubated with HRP-conjugated anti-rabbit or mouse antibody (1:1000, Earth-Ox Life Sciences, Millbrae, CA) for 1 h at room temperature and then exposed and photographed on a Gene Gnome exposure instrument. Finally, the expression of the proteins was standardized for densitometric analysis to β-actin levels.

### Modified neurological severity score test

To evaluate the neurological functional outcomes, the modified neurological severity score (mNSS) test was performed. The tests were carried out before CCI and at days 1, 3, 7 and 14 after CCI. The scale was graded from 0 to 18 (normal score, 0; maximal deficit score, 18). The mNSS comprises the motor (muscle status and abnormal movement), sensory (visual, tactile and proprioceptive) and reflex reactions and balance tests. One point is awarded if the mice are unable to perform the test or lack an expected reaction; thus, the higher the score, the more severe the injury.

### Measurement of the brain water content

Brain oedema was evaluated by measuring the brain tissue water content using the wet-dry weight method as described previously [17]. Animals were sacrificed 7 days after TBI, and the left brain cortical tissue was collected. The brains were harvested, and the pons and olfactory bulbs were removed. The tissue was positioned directly over the injury site, covering the contusion and the penumbra. The fresh tissue was weighed to record the wet weight, dried for 72 h at 80 °C and then weighed again to record the dry weight. The brain water content was calculated using the following formula: (wet weight − dry weight)/wet weight] × 100%.

### Quantitative real-time PCR

Total RNA was isolated from the microglia or tissue using TRIzol (Invitrogen, Carlsbad, CA, USA) before being washed with PBS and reverse-transcribed to cDNA with the PrimeScriptTM RT Reagent Kit (Thermo, USA) according to the datasheet from the manufacturer. Gene products were then amplified by quantitative real-time PCR on an ABI-Prism 7500 Real-Time PCR System (Applied Biosystems, Carlsbad, CA, USA) using SYBR Premix Ex Taq TM II (Takara). The relative level of miR-873a-5p (MQP-0101, RiboBio, China) was normalized to the expression of control U6 snRNA (MQP-0201, RiboBio, China). Other mRNAs were normalized to the internal standard GAPDH. The primers are as follows. Data were analysed using the 2−ΔΔCt method.

**Table:** 

Name	Primer sequence
IL-1β	Forward, AGAACCAAGCAACGACAAAATAC
	Reverse, GTATTGCTTGGGATCCACACTC
IL-6	Forward, GGAGCCCACCAAGAACGATA
	Reverse, CAGGTCTGTTGGGAGTGGTA
TNF-α	Forward, GGATTATGGCTCAGGGTCCA
	Reverse, ACATTCGAGGCTCCAGTGAA
iNOS	Forward, CATTCAGATCCCGAAACGCT
	Reverse, TGTAGGACAATCCACAACTCGC
IL-4	Forward, GTAGGGCTTCCAAGGTGCTTC
	Reverse, CATGATGCTCTTTAGGCTTTCCAG
IL-10	Forward, ACCTGGTAGAAGTGATGCCC
	Reverse, ACACCTTGGTCTTGGAGCTT
Arg1	Forward, GCATATCTGCCAAAGACATCGT
	Reverse, TCTTCCATCACCTTGCCAATC
CD32	Forward, TGTCACTGGGATTGCTGTCG
	Reverse, CCCCAGAGGGCTGTCTGTAC
CD206	Forward, CGTTTCGGTGGACTGTGGA
	Reverse, GTTGTGGGCTCTGGTGGG
GAPDH	Forward, TGAAGGGTGGAGCCAAAAG
	Reverse, AGTCTTCTGGGTGGCAGTGAT

### Immunofluorescence staining

Cells or tissues were incubated with GFAP, Iba1, CD9, Arg1 or iNOS antibodies (Abcam, USA) overnight at 4 °C and then incubated with conjugated secondary antibody for 1 h at room temperature in the dark. After several washes with PBS, the slides were incubated with DAPI for 3 min and then mounted in glycerol. After mounting, immunofluorescent signalling was observed with an Olympus Fluoview laser scanning confocal microscope (Olympus, Tokyo, Japan), and the percentages of positive cells were counted in a blinded manner using ImageJ.

### Statistical analyses

All data are expressed as the mean ± standard deviation. The programmes GraphPad and InStat were used for statistical analyses. One-way ANOVA followed by Newman-Keul’s post hoc test was used for multiple comparisons. A non-paired *t* test was used when two groups were compared. Two-way ANOVA was used to compare the NDs between the three groups at different time points.

## Results

### “Brain extracts” stimulated astrocytes to synthesize and release exosomes

**Fig. 1:**
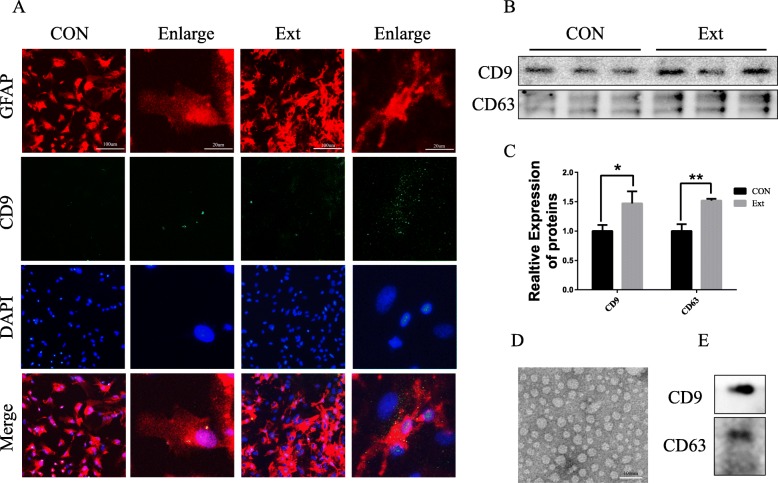
“Brain extracts” stimulated the synthesis and release of exosomes from astrocytes. **a** Exosomes (marked by CD9 (green)) derived from astrocytes were significantly increased under “brain extract” stimulation. **b**, **c** After 24 h of treatment, the medium of primary cultured astrocytes was harvested to detect the markers CD9 and CD63 by Western blotting (data were presented as mean ± SD, *p < 0.05, **p < 0.01, *n* = 5, *t* test). **d** A representative transmission electron microscopy image of purified exosomes from the culture medium of stimulated astrocytes after 24 h of treatment. Scale bar 100 nm. **e** The astrocyte-derived exosomes were identified by the markers CD9 and CD63 by Western blotting

Expression of exosomes in primary cultured astrocytes was detected by immunofluorescence staining. And Western blot was used to analyse exosomes secreted by astrocytes in the medium. Both assays showed that exosomes (marked by CD9 and CD63) increased significantly under stimulation with TBI “brain extract” for 24 h (*p* < 0.05) (Fig. 1a–c). Then, those exosomes were separated from the astrocytes and identified by electron microscopy. TEM imaging showed that the diameter of the exosomes derived from the astrocytes was mostly within the range of 30–100 nm (Fig. 1d), and the marker proteins CD9 and CD63 were also detected (Fig. 1e).

### Astrocyte-derived exosomes are taken up by primary microglia and promote microglial M2 phenotype formation after TBI insult

**Fig. 2:**
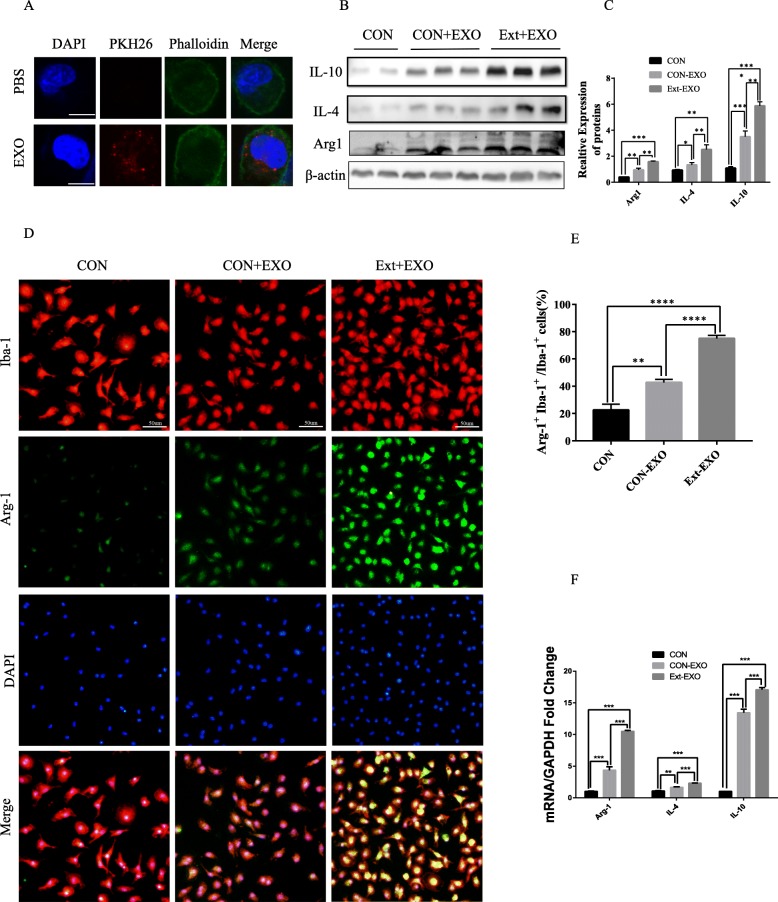
Astrocyte-derived exosomes are taken up by primary microglia and promote microglial M2 phenotype transformation. **a** PKH26 staining and exosome uptake. It was shown by confocal microscopy that exosomes (red) were taken up by the cells and existed in the cytoplasm and around the cell nucleus. Immunofluorescence of the primary microglia showing DAPI (blue), exosomes (red) and F-actin (green). No red fluorescent signal was detected in the PBS control group. PBS: microglial staining with PBS. Exo: the exosomes labelled with pkh26 were incubated with microglia for 24 h. Bar = 10 μm. **b**, **c** Primary cultured microglia were divided into three groups: con, con+Exo and Ext+Exo. Exo: The microglia were incubated with astrocyte-derived exosomes for 4 h. Ext+Exo: primary cultured microglia were stimulated with “brain extracts” for 24 h and washed before incubation with exosomes. These M2 markers were also detected by Western blot analysis. **d**, **e** Fluorescence confocal microscopic images showing both Iba-1 (microglia marker) and Arg-1 (M2 marker) expression increased after astrocyte-derived exosome treatment. Bar = 50 μm. Quantification of the percentage of Arg-1+Iba-1+ cells among total Iba-1+ cells. This effect was more significant after “brain extract” stimulation. **f** The mRNA expression of microglia M2 markers (Arg-1, IL-4, IL-10) was detected by RT-PCR (data were presented as mean ± SD, *p < 0.05, **p < 0.01, ***p < 0.001, *n* = 5, one-way ANOVA)

To determine the effect of astrocyte-derived exosomes on microglial activation, exosomes were separated by an overspeed centrifugation and added to primary cultured microglia. We labelled the exosomes with PKH26 dye (red) to evaluate whether exosomes were taken up by microglia. As showed in Fig. 2a, that exosomes were taken up by microglia. And cell immunofluorescence, qRT-PCR and Western blotting were performed to detect the protein and gene expression of the microglia. The results showed that the exosomes derived from astrocytes can significantly promote microglia into the transformation of the M2 phenotype, and the exosomes derived from the astrocytes after the effects of the brain tissue extracts can further promote this transformation than the normally derived astrocyte exosomes (Fig. 2b–f).

### MicroRNA microarray analysis of exosomes secreted by Ext-stimulated astrocytes

**Fig. 3:**
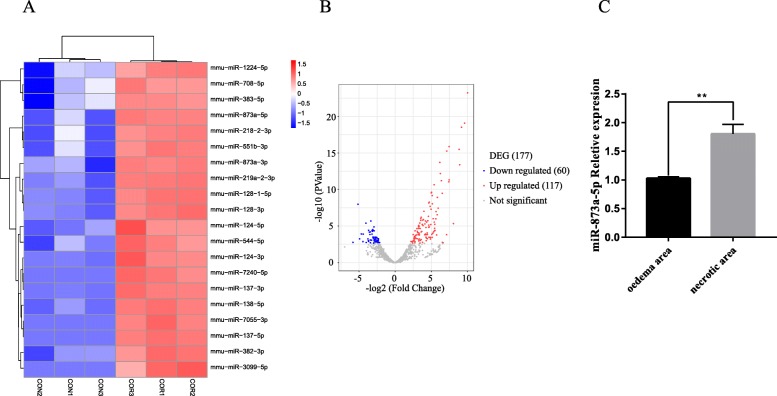
MicroRNA microarray analysis of exosomes released from Ext-stimulated astrocytes. **a** CON: exosomes secreted by astrocytes under physiological conditions. COR: exosomes secreted by astrocytes under simulated trauma. The miRNA component of the exosomes released from stimulated astrocytes was studied by microRNA microarray analysis. The heat map shows the change of the first 20 miRNAs. **b** The miRNA distribution diagram. The transverse axis represents multiple miRNAs in the Ext group compared to those in the CON group, and the longitudinal axis represents the Log10 of the *p* value. **c** The expression of miR-873a-5p in necrotic brain tissue and oedema brain tissue in traumatic brain injury was measured by RT-PCR (data were presented as mean ± SD, compared with the oedema area group, **p < 0.01, *n* = 15, *t* test)

To further explore the active components in astrocyte-derived exosomes, we performed a genome-wide microarray. The results of miRNA microarray analysis of exosomes derived from astrocytes showed that 135 kinds of miRNAs were upregulated significantly (altered more than 2-fold, *p* < 0.05) in the Ext group compared with those in the CON group; the 20 most significantly changed were miR-1224-5p, miR-708-5p, miR-383-5p, miR-873a-5p, miR-218-2-3p, miR-551b-3p, miR-873a-3p, miR-219a-2-3p, miR-128-1-5p, miR-128-3p, miR-124-5p, miR-544-5p, miR-124-3p, miR-7240-5p, miR-137-3p, miR-138-5p, miR-7055-3p, miR-137-5p, miR-382-3p, and miR-3099-5p. Our data (miRNA microarray analysis, Fig. 3a, b) show that miR-873a-5p was in the top 5 changes of all the miRNA in the exosome released by activated astrocytes. Further analysis based on the previous researches and database from the bioinformatics website (http://starbase.sysu.edu.cn) indicates that miR-873a-5p might have an effect on the NF-κB signalling pathway which is well important for microglial activation. Based on the above microarray analysis, we selected miR-873a-5p as the research focus. Brain tissue samples from necrotic and oedema areas were collected 3 days after clinical traumatic brain injury. We detected the expression of miR-873a-5p by qRT-PCR. The results showed that the expression of miR-873a-5p in the lesion area was significantly higher than that in the oedema area (Fig. 3c).

### The effects of miR-873a-5p on primary microglia induced by LPS

**Fig. 4:**
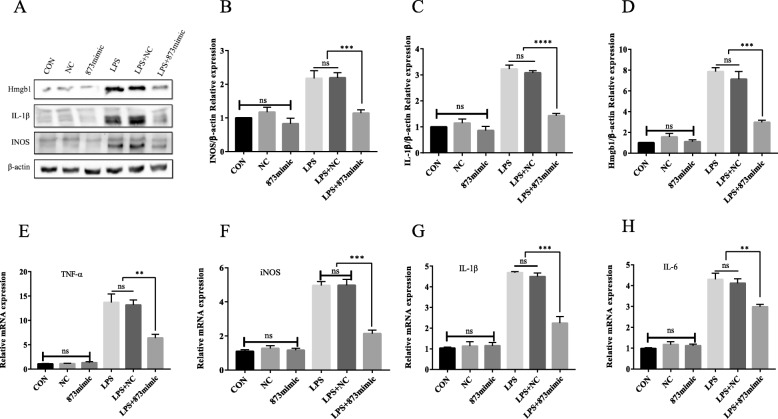
The effects of miR-873a-5p on LPS-activated primary cultured microglia. **a**–**d** Western blotting and statistical analysis of the pro-inflammatory cytokines Hmgb1, IL-1β and iNOS in LPS- and miR-873a-5p-treated cells. **e**–**h** RT-qPCR analyses of changes in the pro-inflammatory cytokines TNF-α, iNOS, IL-1β and IL-6 at the mRNA level (data were presented as mean ± SD, *p < 0.05, **p < 0.01, ***p < 0.001, *n* = 5, one-way ANOVA)

To further explore the function of miR-873a-5p, miR-873a-5p was transfected into primary microglia, which were then stimulated with LPS. We used Western blotting to detect IL-1β, Hmgb1 and INOS protein expression. The mRNA expression of TNF-α, IL-1β, INOS and IL-6 was detected by qRT-PCR. The results showed that compared with the expression levels in the LPS group, the LPS + miR-873a-5p group had significantly inhibited expression of the pro-inflammatory factors IL-1β, INOS, Hmgb1, TNF-α and IL-6, while the LPS + NC group did not have inhibited expression of the pro-inflammatory factors IL-1β, INOS, Hmgb1, IL-6 and TNF-α, as shown in Fig. 4.

### The effects of miR-873a-5p on NF-κB activation in cultured microglia

**Fig. 5:**
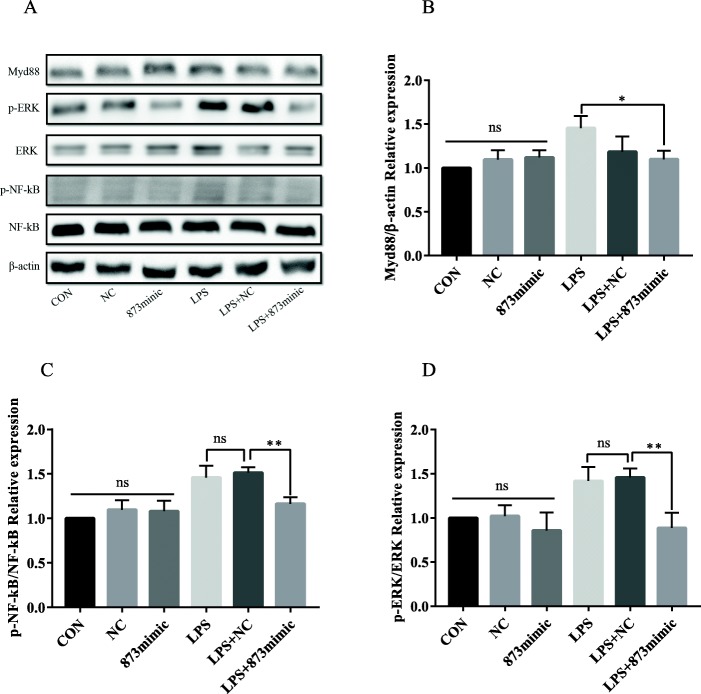
The effects of miR-873a-5p on NF-κB activation in primary cultured microglia. **a**–**d** Western blotting and statistical analysis of Myd88 and phosphorylated NF-κB and ERK (data were presented as mean ± SD, *p < 0.05, **p < 0.01, *n* = 5, one-way ANOVA)

It is well known that NF-κB plays a key role in the regulation of inflammation. To explore how miR-873a-5p inhibits inflammatory responses, we used Western blotting to detect the NF-κB signalling pathway. The results showed that LPS significantly activated the NF-κB signalling pathway in primary microglia, including Myd88, phosphorylated NF-κB and phosphorylated ERK. After intervention with miR-873a-5p, Myd88, phosphorylated NF-κB and phosphorylated ERK were significantly inhibited, while the NF-κB signalling pathway in the LPS + NC group did have dramatic changes (Fig. 5). These results suggest that miR-873a-5p inhibits the LPS-activated NF-κB signalling pathway.

### miR-873a-5p attenuated brain defect area, cerebral oedema content and neurological deficit in mice after TBI

**Fig. 6:**
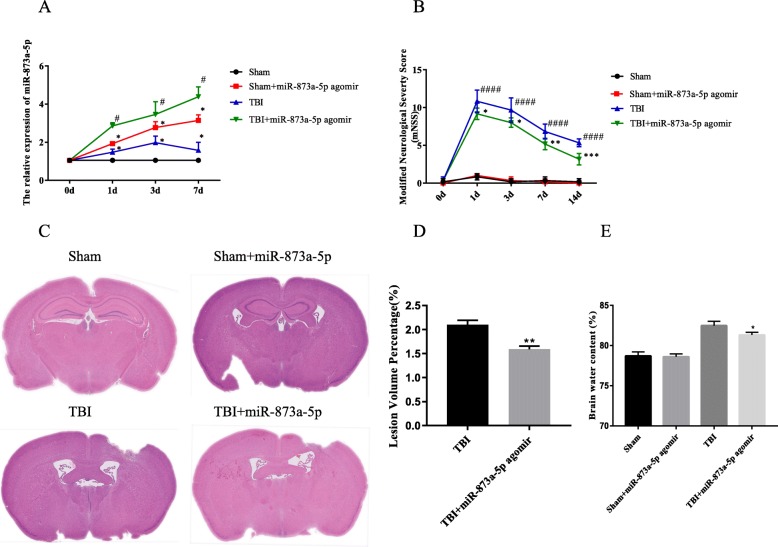
miR-873a-5p attenuated brain defect area, cerebral oedema content and neurological deficit in mice after TBI. **a** miR-873a-5p treatment significantly increased the expression of miR-873a-5p in the cortex at 1, 3 and 7 days after TBI. **b** The nerve function of mice was assessed by mNSS (data were presented as mean ± SD, compared with the sham group *p < 0.05, compared with the TBI group ^#^*p* < 0.05, *n* = 5, one-way ANOVA). **c**, **d** The area of the mice brain defect was determined by the proportional method (data were presented as mean ± SD, compared with the TBI group **p < 0.01, *n* = 5, one-way ANOVA). **e** The water content of brain tissue was measured by the dry-wet method (data were presented as mean ± SD, compared with the TBI group *p < 0.05, *n* = 3, one-way ANOVA)

To identify the effects of miR-873a-5p in traumatic brain injury, TBI was induced in mice by CCI, after which miR-873a-5p was injected into the lateral ventricle. The expression of miR-873a-5p in the cortex was measured by qRT-PCR at 1, 3 and 7 days after TBI. Treatment with miR-873a-5p significantly increased the expression of miR-873a-5p in the cortex at 1, 3 and 7 days after TBI (Fig. 6a). The tissue around the traumatic area of the injured brains and in a similar area of the brains of sham mice was collected. To investigate the neurorestorative effect of miR-873a-5p, we measured the lesion area and cerebral oedema on the seventh day after traumatic brain injury. We also determined a modified neurological score at 7 days after injury. The results showed that the brain damage area, brain oedema degree and neurological function score increased after TBI (Fig. 6b–e). However, treatment with miR-873a-5p significantly reduced the brain damage area and brain oedema degree after TBI on day 7. After miR-873a-5p treatment, the mNSS also improved.

### miR-873a-5p inhibits the inflammatory response by promoting microglial M2 polarization after TBI

**Fig. 7:**
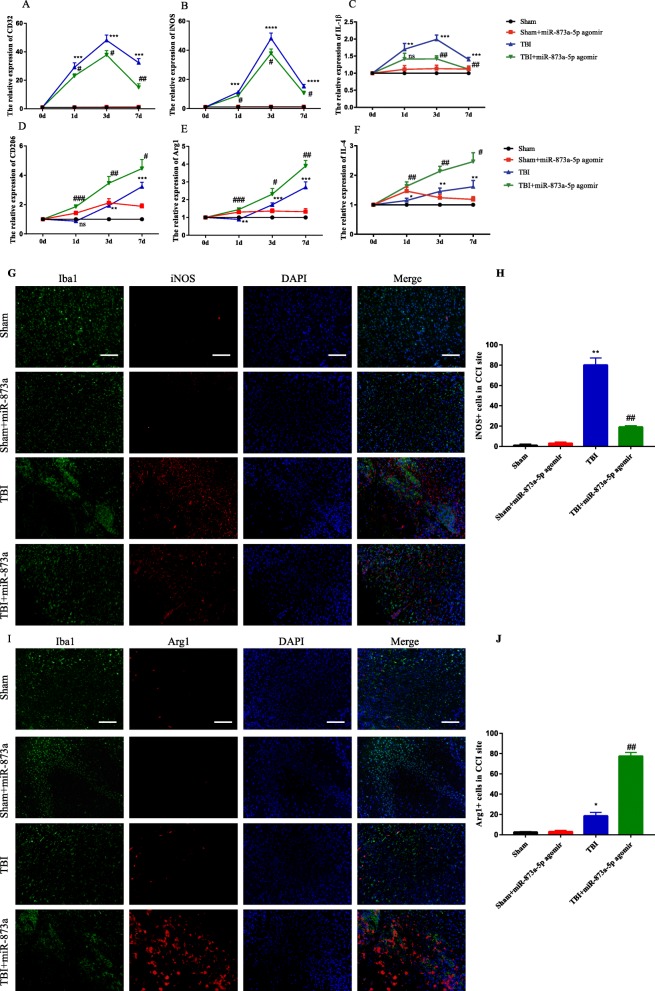
miR-873a-5p treatment inhibits the inflammatory response by promoting microglia polarization to M2 after TBI. **a**–**c** miR-873a-5p treatment significantly reduced the mRNA expression of CD32, INOS and IL-1β in the cortex at 1, 3 and 7 days after TBI. **d**–**f** miR-873a-5p increased the expression of the M2 microglia signature genes CD206, IL4 and Arg1 in the cortex at 1, 3 and 7 days after TBI. **g**, **h** Representative images of double immunofluorescent staining in the injured cortex with the microglial marker Iba1 (green) and the M1 marker iNOS (red). **i**, **j** Representative images of double immunofluorescent staining in the injured cortex with the microglia marker Iba1 (green) and the M2 marker Arg1 (red). Bar = 50 μm (data were presented as mean ± SD, compared with the sham group *p < 0.05, **p < 0.01, ***p < 0.001; compared with the TBI group ^#^*p* < 0.05, ^##^*p* < 0.01, ^###^*p* < 0.001, *n* = 5, one-way ANOVA)

To specifically evaluate the polarization state of microglia after TBI, qRT-PCR was performed to measure the polarization of microglia in the cortex by detecting the expression of the M1 signature genes INOS, CD32 and IL-1β and the M2 signature genes CD206, IL-4 and Arginase 1. The results demonstrated that the expression of the M1 signature genes CD32, INOS and IL-1β and the expression of the M2 signature genes CD206, IL-4 and Arg1 were significantly elevated in the cortex at 1, 3 and 7 days after TBI. miR-873a-5p treatment significantly reduced the expression of CD32, INOS and IL-1β (Fig. 7a–c) in the cortex at 1, 3 and 7 days after TBI. Furthermore, the miR-873a-5p treatment also significantly increased the expression of CD206, IL-4 and Arg1 (Fig. 7d–f) in the cortex at 1, 3 and 7 days after TBI. Moreover, the representative M1-associated marker (INOS) or M2-associated marker (Arg1) proteins were double-labelled with Iba1, a microglia marker, in traumatic foci in the injuredcortex. An immunofluorescence study revealed that the expression of INOS increased in the injured cortex (Fig. 7g, h) at day 3 post-injury and reached peak levels on day 3 but was significantly decreased by upregulating miR-873a-5p. The overexpression of miR-873a-5p increased the number of cells labelled with the M2 marker Arg1 in the injured cortex (Fig. 7i, j). These findings were consistent with the qRT-PCR results (Fig. 7b, e). Taken together, these findings demonstrated that upregulating miR-873a-5p expression significantly alters the M1/M2 phenotype balance by inhibiting M1 activation and enhancing M2 activation after TBI.

### The in vivo effects of miR-873a-5p on NF-κB activation in the mice TBI model

**Fig. 8:**
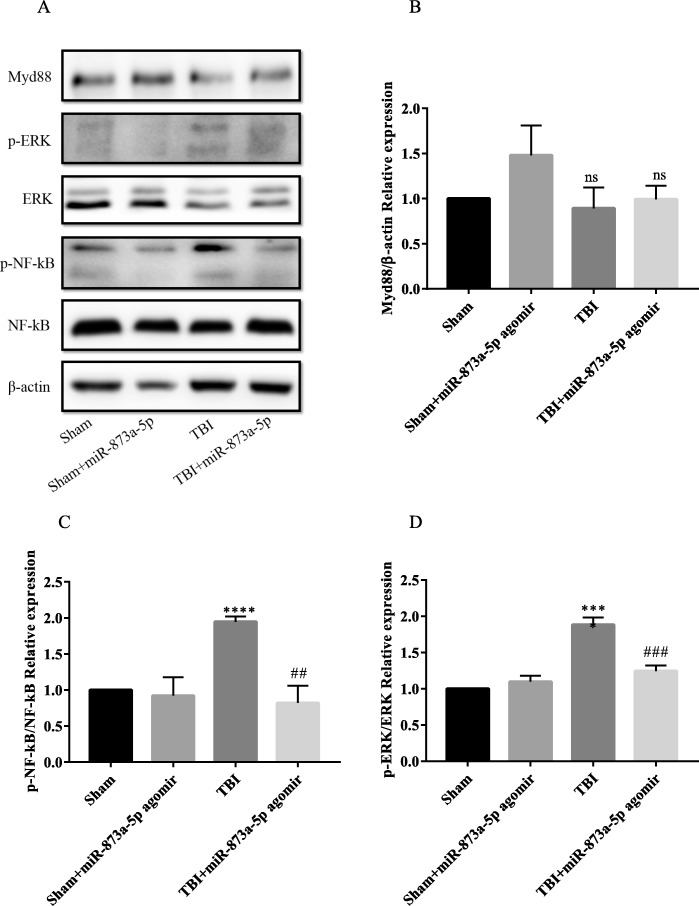
The in vivo effects of miR-873a-5p on NF-κB activation in mice TBI model. **a**–**d** Brain tissue of the in vivo mice TBI model was collected at the seventh day post-TBI. Western blot demonstrating that miR-873a-5p agomir administration suppressed Myd88 signal activation and reversed TBI-induced phosphorylation of NF-κB and ERK (data were presented as mean ± SD, compared with the sham group ****p < 0.0001, compared with the TBI group ^##^*p* < 0.01, ^###^*p* < 0.001, *n* = 5, one-way ANOVA)

We evaluated Myd88, ERK and NF-κB p65 expression via WB analysis to investigate the cellular mechanisms by which miR-873a-5p inhibits the NF-κB signalling pathway after TBI. The tissue was collected at day 7 post-TBI because the NF-κB signalling pathway was inhibited effectively at this time point. The expression of Myd88 and phosphorylated ERK and NF-κB p65 was relatively low in cerebral samples of the sham mice, whereas Myd88 and phosphorylated ERK and NF-κB p65 levels were substantially increased in brain tissue collected from the TBI mice (Fig. 8). Compared to the expression in the TBI group, the expression of Myd88 and phosphorylated ERK and NF-κB p65 was dramatically decreased in the TBI + miR-873a-5p group (Fig. 8).

## Discussion

In this study, we determined that activated astrocytes can regulate microglial polarization by releasing miRNAs that are transported by exosomes in mice TBI models in vitro and in vivo. Although the exosomes derived from BE-stimulated astrocytes require further exploration, our findings are still consistent with evidence that (1) BE-stimulated astrocyte-derived exosomes were enriched with microRNA-873a-5p, which can promote microglial M2 phenotype transformation in the early stage of TBI; (2) miR-873a-5p effectively suppressed pro-inflammatory factors and promoted anti-inflammatory factors released from the microglia via inhibiting the phosphorylation of ERK and the NF-κB signalling pathway; and (3) the anti-neuroinflammatory effect of miR-873a-5 can attenuate the mNSS and brain oedema in mice TBI model.

Although the microglia play a key role in the inflammatory and immune response following TBI, there are still no effective therapeutic targets to regulate their activation and function. Recent evidence showed that the microglia acted as a double-edged sword in many pathophysiological conditions based on their differential polarization: the pro-inflammatory M1 and anti-inflammatory M2 phenotypes. This M1/M2 phenotype transformation can also be observed in neuroinflammation following TBI. Our previous study indicated that promoting microglial transformation from the M1 to M2 phenotype is beneficial for attenuating the immune response and ameliorating neurological impairment after brain trauma [14]. However, up to now, the regulation of the microglial phenotype is lacking as a specific effective drug treatment.

Some research declares that astrocytes have a powerful ability to regulate microglial activation. A number of studies indicate that astrocytes in the brain are active participants in both propagating and regulating neuroinflammation [8]. Astrocytes become activated by inflammatory mediators and cytokines and produce many regulatory factors that may influence CNS immunity. It was reported that proinflammatory cytokines such as TNFα and IL-1β may stimulate astrocytes toward a predominantly harmful reactive state (A1). Meanwhile, stimulation with the anti-inflammatory cytokine IL-4 can lead to a more protective or restorative reactive state A2 [18, 19]. The results showed that both A1 phenotype biomarker (iNOS and TNF-α) and A2 biomarker (TGF-β and Arg-1) were increased after TBI (additional figure 1). In our previous study (the manuscript under submission), IL-1β and IL-4 were upregulated in the “brain extracts”. This may explain why both the A1 and A2 astrocyte phenotypes existed. It has been reported that the addition of conditioned media from astrocytes to microglia cultures increased antioxidant and anti-inflammatory gene expression by providing negative feedback to activated microglia [9]. However, the underlying mechanism has not been well defined. Some recent studies proposed that exosomes take part in cell-to-cell communication. For example, Hu et al. demonstrated that EVs derived from astrocytes exposed to morphine can be taken up by microglial endosomes, leading, in turn, to activation of Toll-like receptor 7 (TLR7) [20]. Sobue et al. reported that the overexpression of MHCI in astrocytes affects microglial proliferation as well as neuronal numbers and spine densities, thereby leading to social and cognitive deficits in mice, possibly via exosomes created by astrocytes [21]. However, there are few reports about the immunoregulatory effects of exosomes from astrocytes on microglial activation. The highlight of this study is that we used BE to stimulate primary cultured astrocytes to simulate traumatic brain injury in vivo. The results confirmed that stimulated astrocytes produce and secrete more exosomes that promote the microglia phenotype transformation from M1 to M2.

The cargo shuttled by exosomes is complicated, including proteins, miRNAs, and mRNAs. We focused on miRNAs whose potential effects have been increasingly appreciated. Recently, some astrocyte-derived miRNAs have been investigated. Xu et al. reported that miR-92b-3p from preconditioned astrocytes can be carried to neurons by exosomes and ameliorate OGD-induced cell death and apoptosis [22]. miR-7 from astrocytes can cause the downregulation of neuronal neuroligin 2 (NLGN2) and ultimately lead to synaptic alterations after HIV-1 infection [23]. Through high-throughput miRNA sequencing, we found that the expression of miR-873a-5p in exosomes was significantly changed after BE insult. This microRNA exhibited anti-inflammatory effects by suppressing LPS-induced microglial activation. This is the first report to demonstrate the function of miR-873a-5p in regulating inflammation during brain trauma.

As a tumour-associated miRNA, miR-873a-5p has been well recognized to regulate tumour proliferation and invasion by modulating certain molecules in colorectal cancer, endometrial cancer and hepatocellular carcinoma [24–26]. Cui et al. reported that the expression level of miR-873a-5p in breast tumours was much lower than normal. Moreover, the overexpression of miR-873a-5p or its mimic inhibits breast cancer cell proliferation both in vitro and in an in vivo mouse model [27]. It has been established that miR-873 increases lung adenocarcinoma cell proliferation and migration by targeting SRCIN1, and its expression is decreased in glioblastoma multiforme (GBM) tumour tissues and cell lines [28]. Liu et al. first reported that miR-873 in astrocytes induced by IL-17 promotes inflammatory cytokine production and exacerbates demyelination in experimental autoimmune encephalomyelitis (EAE) through the A20/NF-κB pathway [29]. However, the functional subtype of miR-873 and its correlation with microglia are unclear. Our results indicated that miR-873a-5p expression was increased, and release by activated astrocytes might be a negative feedback mechanism to the microglia-mediated immune response during TBI insult. This might be a potent treatment target for regulating neuroinflammation. This phenomenon is also confirmed by LPS-stimulated microglia in vitro. These data are inconsistent with the results reported by Liu’s group. The possible reasons are as follows: Liu et al. did not indicate which subtype of miR-873 plays a pro-inflammatory role. Also, miR-873a may have a pro-inflammatory effect on astrocytes and an anti-inflammatory effect on microglia, which may also explain the inflammatory balance in the central nervous system. These data together demonstrate a new role for miR-873a-5p in regulating microglial activation. The inflammatory response is the main cause of damage after TBI, and miR-873 can exert neuroprotective effects by inhibiting this inflammation.

NF-κB is a classical inflammatory signalling pathway that is associated with neurotoxicity [30]. This study showed that the NF-κB signalling pathway was likely one of the important targets of miR-873a-5p. Suppression of the NF-κB signalling pathway with miR-873a-5p could transform microglial polarization from M1 to M2 under TBI conditions. This effect of miR-873a-5p can provide new strategies for future clinical drug development.

## Conclusion

Taken together, our results showed that astrocyte-derived exosomes enriched with miR-873a-5p attenuated neurological deficits post-TBI by promoting microglial polarization into the M2 phenotype via inhibiting ERK and NF-κB p65 phosphorylation. These findings suggest that miR-873a-5p might be a potent therapeutic target for ameliorating cerebral injury and improving neurological function after TBI.

## Supplementary information

**Additional file 1: Fig 1.** “Brain extracts” activated astrocytes. CON: astrocytes under physiological conditions. COR: astrocytes under simulated trauma. (A-B) The mRNA expression of astrocytes A1 markers (INOS, TNF-α) was detected by qRT-PCR. (C-D) The mRNA expression of astrocytes A2 markers (Arg-1, TGF-β) was detected by qRT-PCR. (The values are expressed as the mean ± standard deviation: *p < 0.05, **p < 0.01, *n* = 5, t-test.).

**Additional file 2: Table 1.** The 15 clinical patients’ information.

## Data Availability

The datasets used and/or analysed during the current study are available from the corresponding author on reasonable request.

## Abbreviations

Arg1: Arginase-1
CCI: Controlled cortical impact
CD: Cluster of differentiation
ERK: Extracellular regulated protein kinases
EXO: Exosome
Ext: Brain extracts
GAPDH: Glyceraldehyde-3-phosphate dehydrogenase
Hmgb1: High-mobility group protein
Iba1: Ionized calcium-binding adaptor molecule 1
ICV: Intracerebroventricular infusion
IL: Interleukin
iNOS: Inducible nitric oxide synthase
LPS: Lipopolysaccharide
mNSS: Modified neurological severity score
MyD88: Myeloid differentiation factor 88
NF-κB: Nuclear factor-κB
qRT-PCR: Quantitative real-time polymerase chain reaction
TBI: Traumatic brain injury
TLR: Toll-like receptor
TNF-α: Tumour necrosis factor-α
