# Supplementary figures and images for: Astrocyte-derived exosomes enriched with miR-873a-5p inhibit neuroinflammation via microglia phenotype modulation after traumatic brain injury
Source: J Neuroinflammation. 2020 Mar 19;17:89. doi: 10.1186/s12974-020-01761-0 (PMC7082961; doi:10.1186/s12974-020-01761-0)

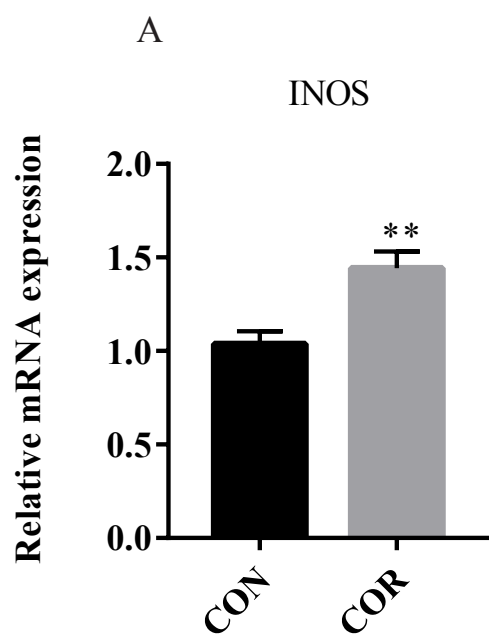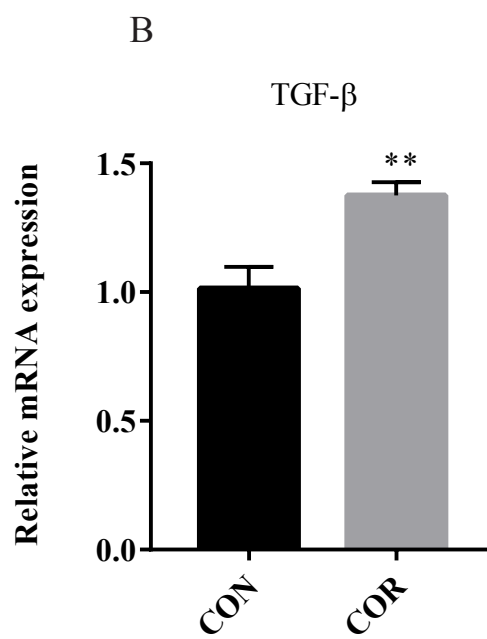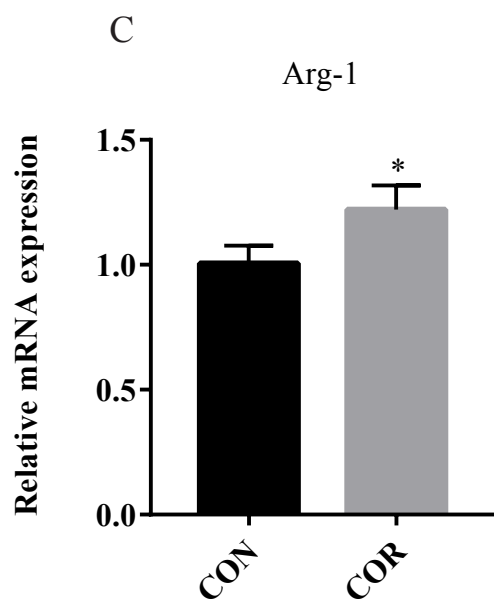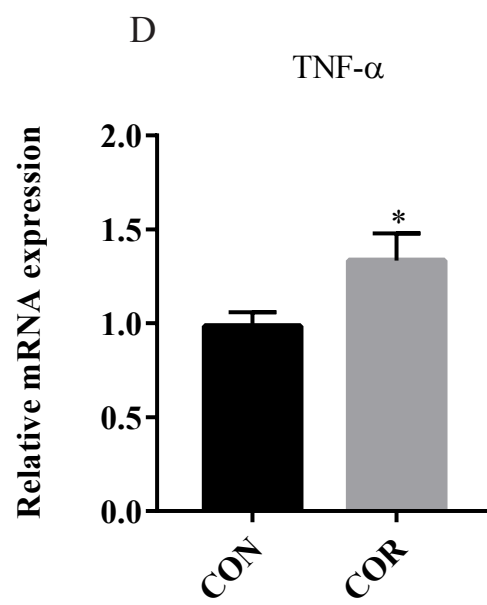

Supplement: Supplementary file 1 — Additional file 1: Fig 1. “Brain extracts” activated astrocytes. CON: astrocytes under physiological conditions. COR: astrocytes under simulated trauma. (A-B) The mRNA expression of astrocytes A1 markers (INOS, TNF-α) was detected by qRT-PCR. (C-D) The mRNA expression of astrocytes A2 markers (Arg-1, TGF-β) was detected by qRT-PCR. (The values are expressed as the mean ± standard deviation: *p < 0.05, **p < 0.01, n = 5, t-test.). [file 12974_2020_1761_MOESM1_ESM.pdf]
