# Supplementary material for: Astrocyte-derived exosomes enriched with miR-873a-5p inhibit neuroinflammation via microglia phenotype modulation after traumatic brain injury
Source: J Neuroinflammation. 2020 Mar 19;17:89. doi: 10.1186/s12974-020-01761-0 (PMC7082961; doi:10.1186/s12974-020-01761-0)
Supplement: Supplementary file 2 — Additional file 2: Table 1. The 15 clinical patients’ information. [file 12974_2020_1761_MOESM2_ESM.pdf]

Additional table 1. The 15 clinical patients' information.

Patient ID	Gender	Age	Region of the brain	Glasgow coma scale
01	Male	58	Right frontal lobe	6
02	Male	48	Left frontal lobe	5
03	Male	38	Left frontal lobe	6
04	Female	49	Left frontal lobe	5
05	Male	35	Right frontal lobe	6
06	Male	54	Left frontal lobe	5
07	Male	36	Right frontal lobe	5
08	Female	37	Left frontal lobe	7
09	Male	42	Right frontal lobe	7
10	Male	46	Right frontal lobe	6
11	Male	54	Right frontal lobe	7
12	Female	57	Left frontal lobe	6
13	Male	38	Right frontal lobe	5
14	Male	41	Right frontal lobe	6
15	Male	39	Left frontal lobe	7
